# Relationship between dietary intake, eating attitudes, and premenstrual syndrome severity among Iranian women: insights from a cross-sectional study

**DOI:** 10.1186/s40337-025-01326-7

**Published:** 2025-07-10

**Authors:** Sara Mighani, Fatemeh Taghizadeh Shivyari, Alireza Razzaghi, Mohammad Amerzadeh, Maryam Javadi

**Affiliations:** 1 Student Research Committee, School of Public Health, Qazvin University of Medical Sciences, Qazvin, Iran; 2 Social Determinants of Health Research Center, Research Institute for Prevention of Non- Communicable Diseases, Qazvin University of Medical Sciences, Qazvin, Iran; 3 Children Growth Research Center, Research Institute for Prevention of Non-Communicable Diseases, Qazvin University of Medical Sciences, Qazvin, Iran

**Keywords:** Premenstrual syndrome, Dietary factors, PMS, Eating disorder

## Abstract

**Background:**

Premenstrual syndrome (PMS) is a common issue that impacts many women, and a well-balanced diet can help alleviate PMS symptoms. Evidence suggests that dietary factors and eating disorders may influence PMS severity, yet the specific relationships remain unclear. This study aimed to evaluate the association of specific dietary components and eating behaviors with PMS symptoms.

**Methods:**

This cross-sectional study was conducted on 252 women with PMS who were referred to healthcare centers in Qazvin province. Data concerning PMS, dietary factors, and eating disorders were collected using online questionnaires, including the premenstrual symptoms screening tool (PSST), 3-day dietary recall, and Eating Attitudes Test-26 (EAT-26) questionnaires. Statistical analysis utilized ANOVA and chi-square tests. The adequacy of the sample was evaluated using the Kaiser-Meyer-Olkin (KMO) test. To examine the relationship between eating disorders, dietary factors, and PMS symptoms, multivariable linear regression analysis was conducted. Statistical significance was set at *p* < 0.05.

**Results:**

According to the PSST, 28.7% of individuals reported mild severity, 21.9% reported moderate severity, and 49.4% reported severe severity. The study revealed that higher sodium (*p* = 0.003, OR = 1.000, 95% CI = [1.000, 1.001]), vitamin D (*p* = 0.044, OR = 1.298, 95% CI = [1.007, 1.674]), and vitamin C intake were positively linked to increased psychological PMS symptoms, (*p* = 0.036, OR = 1.006, 95% CI = [1.000, 1.012]) while magnesium showed a negative association with these symptoms. Also, sodium and vitamin D intake were significantly associated with increased physical symptoms (*p* < 0.05). Individuals with eating disorders showed higher rates of severe PMS symptoms, though differences were not statistically significant (*p* > 0.05).

**Conclusion:**

These findings highlight the potential influence of specific nutrients on PMS severity. This insight could inform dietary recommendations for managing PMS symptoms, providing young women with potential non-pharmacological options to relieve discomfort.

## Abstract (plain language summary)

This study aimed to evaluate the association of specific dietary components and eating behaviors with PMS symptoms. This study was conducted on 252 women with PMS in Qazvin, Iran. According to the PSST, 49.4% of individuals reported severe severity. The study revealed that higher sodium, vitamin D, and vitamin C intake were positively linked to increased psychological PMS symptoms, while magnesium showed a negative association with these symptoms. Also, sodium and vitamin D intake were significantly associated with increased physical symptoms. These findings highlight the potential influence of specific nutrients on PMS severity. This insight could inform dietary recommendations for managing PMS symptoms, providing young women with potential non-pharmacological options to relieve discomfort.

## Background

Premenstrual syndrome (PMS) is a prevalent health concern affecting a significant proportion of women of reproductive age. It is characterized by various physical and emotional symptoms, including mood swings, irritability, fatigue, and bloating, which can significantly impair quality of life and daily functioning [1]. The prevalence of PMS in the Iranian population was reported at 70.8% overall, which was higher (80.4%) in high school students [2]. While the precise etiology of PMS remains unclear, hormonal fluctuations, particularly in estradiol and progesterone levels, during the luteal phase are believed to influence appetite, food cravings, and dietary intake patterns [3, 4]. Increasing evidence suggests that dietary factors may modulate PMS symptoms, yet findings across studies remain inconsistent.

While some research has found associations between high intake of processed foods and more severe PMS symptoms [5], other studies report no significant correlations [6]. Conversely, diets rich in fruits, vegetables, calcium, magnesium, vitamin B6, and omega-3 fatty acids have been associated with reduced PMS symptoms, while excessive sodium and caffeine intake may aggravate them [7, 8]. Recent studies have further explored the role of overall dietary patterns, such as the DASH diet and carbohydrate quality index, in relation to PMS symptoms [9, 10]. Beyond dietary components, eating disorders may also play a role in the severity of PMS symptoms.

Disorders such as anorexia nervosa, bulimia nervosa, and binge-eating disorder can alter nutrient intake and hormonal balance, potentially affecting PMS [11].

Eating disorders are one of the mental illnesses that is growing worldwide. The intricate relationship between diet and PMS is further complicated by the prevalence of eating disorders among women. Conditions such as anorexia nervosa, bulimia nervosa, and binge-eating disorder can disrupt nutrient intake and overall health, potentially influencing the severity of PMS symptoms [12].

A study by Yi et al. demonstrated a positive correlation between the severity of eating disorders and PMS, highlighting the importance of considering these factors in the management of PMS [13]. On the other hand, the study by Bahadur found a weak relationship between the high prevalence of PMS and eating disorder [14].

However, few studies have simultaneously examined the interaction between dietary factors and eating behaviors in the context of PMS.

This study seeks to address this gap by investigating the combined effects of specific nutrients, dietary habits, and eating disorders on PMS symptom severity.

By clarifying these relationships, we aim to contribute new insights that may inform holistic, non-pharmacological strategies for the prevention and management of PMS.

## Material and method

### Study design and participants

This cross-sectional survey was conducted between 2022 and 2023 using an online questionnaire completed by females of reproductive age who were referred to three health centers in Qazvin, Iran. For this purpose, we sent an invitation message to the required number of people. In this invitation letter, the objectives of the participation plan were explained, and at the end of it there was a Google Form link for the questionnaires. If the participants agree and are satisfied, they click on the link and complete the questionnaire. A total of 252 women participated and completed the questionnaire based on specific inclusion criteria. Participants were selected by healthcare professionals and were required to have a regular menstrual cycle. The inclusion criteria specified that participants must be in reproductive age, willing to participate, and experiencing PMS symptoms. Women who were pregnant, breastfeeding, or menopausal were excluded. Additionally, participants with diagnosed diseases such as diabetes, liver or kidney dysfunction, cardiovascular disease, cancer, polycystic ovary syndrome, or premature menopause, as well as those taking birth control pills, antidepressants, thyroid medication, and other hormonal treatments were not eligible. This study was approved by the Ethics Committee of Qazvin University of Medical Sciences (Ethics Code: IR.QUMS.REC.1401.110). All participants provided informed consent by completing the questionnaire and their information was kept confidential.

### Methodological quality

The methodological quality of the study was evaluated using the STROBE checklist [15] to ensure robust reporting of observational studies. Participants were first screened based on inclusion and exclusion criteria. Eligible women were informed about the study objectives and were provided with a link to the online questionnaire via Google Form. The data collection process involved three tools: a demographic questionnaire, 3-day dietary recall, the Eating Attitudes Test (EAT-26), and the Premenstrual Symptoms Screening Tool (PSST).

### Eating attitudes test (EAT-26)

The EAT-26 is a self-report questionnaire to evaluate attitudes and behaviors related to eating and body image, particularly in individuals at risk for eating disorders. Comprising 26 items, the test assesses items on a scale that ranges from never (0 points), rarely (0 points), sometimes (0 points), often (1 points), usually (2 points), and always (3 points) for questions 1 to 25 and question 26, never (3 points), rarely (2 points), sometimes (1 points), often (0 points), usually (0 points), and always (0 points). Scores are summed to determine the level of concern and higher scores indicating more problematic attitudes. The total score can range from 0 to 78. Score 0–20 indicates healthy eating attitudes and behaviors. Score 21–30 shows some concerning attitudes related to eating. Score 31 and above suggest significant concerns related to eating and body image. The Persian version of EAT-26 has demonstrated good internal consistency, with a Cronbach’s alpha of 0.76–0.92 [16].

### Premenstrual symptoms screening tool (PSST)

The PSST is a 19-item valid [17] tool divided into two domains. The reliability of the Iranian version of the PSST has been confirmed with a Cronbach’s alpha of 0.93. The first domain comprises 14 items that focus on psychological, physical, and behavioral symptoms, while the second domain consists of five items that assess how these symptoms affect women’s functioning. Each item is scored on a four-point scale: not at all = 0, mild = 1, moderate = 2, and severe = 3.

### Three-day dietary recall (3-day recall)

To gather comprehensive information regarding all foods and beverages consumed within a specific day, a dietary recall was conducted for three days, two week days, and one day in weekend. Due to the large sample size and the nature of online data collection, it was not feasible to conduct in-person interviews required for more detailed tools such as the food frequency questionnaire (FFQ). Therefore, the 3-day dietary recall method was chosen as a practical alternative for assessing dietary intake in this context. Although this questionnaire is a widely used method for dietary assessment, it has some inherent limitations, such as recall bias and the inability to reflect seasonal or daily variations in food intake. However, due to its practicality, low cost, and ability to provide reasonably accurate estimates of usual short-term intake, especially in large cross-sectional studies, it was considered an appropriate tool for this research. This method is commonly used in Iranian nutritional studies and has been validated by experts in previous research [18]. The data on food recall was analyzed by Nutritionist-4 software.

### Sample size calculation

Based on a previous study by Mona S. Hashim et al., the prevalence of moderate to severe PMS was estimated to be 63% [19]. Using a 95% confidence level (α = 0.05) and a margin of error of 4%, the required sample size was calculated to be 560 participants.

$$n=\frac{Z^{2}.P(1-P)}{d^{2}}$$

### Data analysis

Data were analyzed using SPSS version 20. Quantitative variables were assessed using ANOVA, while qualitative variables were evaluated using chi-square tests. Sampling adequacy was verified through the Kaiser-Meyer-Olkin test. The results of the factor analysis are provided in the Appendix. Factors with eigenvalues greater than 1 were considered significant. To examine the relationship between eating disorders, dietary factors, and PMS symptoms, multivariable linear regression analysis was conducted. Statistical significance was set at *p* < 0.05.

## Results

After distributing the questionnaire to 560 women from health centers, 252 met the entry criteria and were diagnosed with PMS using the PSST questionnaire. However, some participants did not fully complete the questions related to PMS, resulting in 237 valid responses for analysis. This discrepancy is due to the fact that the questionnaire was distributed to 560 women; however, many responses were either incomplete, did not meet the inclusion criteria, or did not indicate PMS according to the PSST. Thus, only the complete and eligible cases were analyzed in the study. The demographic characteristics of the participants are presented in Table 1. The mean age of the participants was 32.41 ± 10.18 years. Among them, 40.1% were married, while 59.9% were single. Regarding educational background, 3.6% of the participants had completed high school, whereas 95.7% had obtained higher education. In terms of employment status, 33.3% were employed, 50% were students, and 16.3% were homemakers. The mean ± standard deviation (SD) of participants’ weight was 62.56 ± 11.49 kg, while the mean ± SD of the number of family members was 3.88 ± 11.2.

**Table 1:** Participant baseline characteristics

Characteristics		*N* = 252 (%)
Age; mean ± SD (range) years 32.41 ± 10.18		
Marriage status	Married	101 (40.1%)
	Single	151 (59.9%)
Education	High school	9 (3.6%)
	Diploma and postgraduate diploma	45 (17.9%)
	Bachelor’s degree	101 (40.1%)
	Master’s degree	69 (27.4%)
	PhD and above	26 (10.3%)
Occupational status		
	Employee	85 (33.3%)
	Student	126 (50.0%)
	Housewife	41 (16.3%)
Weight; mean ± SD		62.56 ± 11.49
Number of family members; mean ± SD		3.88 ± 11.2

The classification of PMS severity among participants, as assessed using the Premenstrual Symptoms Screening Tool (PSST) questionnaire, is summarized in Table 2. Based on the PSST results, 28.7% of participants experienced mild PMS, 21.9% had moderate PMS, and 49.4% reported severe PMS symptoms.

**Table 2:** Classification of disease severity in patients according to PMS symptoms

Severity of PMS symptoms	PSST Frequency	Percent (%)
Mild	68	28.7
Moderate	52	21.9
Severe	117	49.4

Table 3 presents the detailed frequency and valid percentages of PMS symptoms. Distribution of the severity of PMS symptoms among participants, categorized into four levels (severe, moderate, mild, and none). The majority of symptoms were reported as severe. The highest intensity for each symptom is highlighted in bold within the table to allow easier identification of the more common symptoms.

**Table 3:** Frequency and percentage of PMS symptoms according to PSST domains

Items	Never Frequency (valid percentage)	Mild Frequency (valid percentage)	Moderate Frequency (valid percentage)	Severe Frequency (valid percentage)
Anger/irritability	27 (10.8)	65 (25.9)	**104 (41.4)**	55 (21.9)
Anxiety/tension	36 (14.3)	67 (26.7)	**109(43.4)**	39 (15.5)
Tearful/sensitive to rejection	55 (21.9)	70 (27.9)	**84 (33.5)**	42 (16.7)
Depressed mood/hopelessness	47 (18.7)	**77 (30.6)**	75 (29.8)	53 (21)
Decreased interest in work activities	68 (27)	**85 (33.7)**	70 (27.8)	29 (11.5)
Decreased interest in home activities	55 (21.8)	**85 (33.7)**	71 (28.2)	41 (16.3)
Decreased interest in social activities	73 (29)	**78 (31)**	74 (29.4)	27 (10.7)
Difficulty concentrating (such as not concentrating on a lesson or any other task)	67 (26.6)	**86 (34.1)**	70 (27.8)	29 (11.5)
Fatigue/lack of energy	16 (6.4)	**83 (33.1)**	93 (37.1)	59 (23.5)
Overeating/ food cravings	66 (26.2)	**77 (30.6)**	79 (31.3)	30 (11.9)
Insomnia	**115 (45.8)**	79 (31.5)	43 (17.1)	14 (5.6)
Hypersomnia (need more sleep)	66 (26.2)	70 (27.8)	**88 (34.9)**	28 (11.1)
Feeling overwhelmed or out of control	59 (23.5)	**84 (33.5)**	75 (29.9)	33 (13.1)
Physical symptoms such as breast pain, headache, muscle/joint pain, abdominal bloating, weight gain	27 (10.8)	67 (26.7)	**97 (38.6)**	60 (23.9)
Work efficiency/productivity	72 (28.8)	**85 (34)**	81 (32.4)	12 (4.8)
Relationship with co-workers	65 (25.8)	**89 (35.3)**	77 (30.6)	21 (8.3)
Relationship with family	55 (22)	**88 (35.2)**	84 (33.6)	23 (9.2)
Social life activities	73 (29)	**92 (36.5)**	72 (28.6)	15 (6)
Home Responsibilities	63 (25.1)	**90 (35.9)**	76 (30.3)	22 (8.8)

Before conducting the logistic regression analysis, the main assumptions including linearity of the logit for continuous variables, absence of multicollinearity, and approximate normality of residuals were generally assessed and considered acceptable. This study examined the associations between dietary factors and PMS symptoms, categorized into psychological, physical, and behavioral domains, as well as the relationship between eating disorders and PMS. Table 4 provides a summary of the associations between dietary factors and PMS symptoms across these domains.

### Psychological symptoms

Among the dietary factors analyzed, sodium intake exhibited a significant positive association with psychological symptoms (*p* = 0.003, OR = 1.000, 95% CI = [1.000, 1.001]). In contrast, magnesium intake showed a significant negative association (*p* = 0.009, OR = 0.992, 95% CI = [0.987, 0.998]). Additionally, vitamin C (*p* = 0.036, OR = 1.006, 95% CI = [1.000, 1.012]) and vitamin D (*p* = 0.006, OR = 1.406, 95% CI = [1.105, 1.788]) demonstrated significant positive associations with psychological symptoms.

### Physical symptoms

Sodium intake was also significantly positively associated with physical symptoms (*p* = 0.003, OR = 1.000, 95% CI = [1.000, 1.001]). Moreover, vitamin D intake showed a significant positive relationship with physical symptoms (*p* = 0.044, OR = 1.298, 95% CI = [1.007, 1.674]).

### Behavioral symptoms

Behavioral symptoms exhibited marginal associations with certain dietary factors. Caloric intake was inversely associated with behavioral symptoms (*p* = 0.069, OR = 0.976, 95% CI = [0.952, 1.002]). In contrast, saturated fat intake (*p* = 0.076, OR = 1.612, 95% CI = [0.951, 2.732]) and carbohydrate intake (*p* = 0.054, OR = 1.108, 95% CI = [0.998, 1.229]) showed positive trends, suggesting a potential exacerbation of symptoms with higher intake. However, none of these associations reached statistical significance (*p* > 0.05).

These findings highlight the role of specific nutrients, such as sodium, magnesium, and vitamins C and D, in influencing PMS symptoms.

The relationship between eating disorders and PMS symptoms is presented in Table 5. Individuals with eating disorders exhibited a higher prevalence of severe symptoms across psychological (18.6%), physical (19%), and behavioral (12.5%) domains compared to those without eating disorders. However, these associations were not statistically significant (*p* > 0.05).

**Table 4:** Association between dietary factors and PMS symptoms

Variable		Psychological symptoms			Physical symptoms				Behavioral symptoms			
	P	OR	95% CI		P	OR	95% CI		P	OR	95% CI	
Energy (kcal)	0.240	1.009	0.994–1.023		0.435	0.994	0.980–1.009		0.069	0.976	0.952–1.002	
Cho (g)	0.278	0.968	0.913–1.026		0.552	1.019	0.962–1.097		0.054	1.108	0.998–1.229	
Protein (g)	0.360	0.971	0.910–1.035		0.425	1.026	0.963–1.093		0.124	1.101	0.974–1.245	
Fat (g)	0.264	0.916	0.785–1.069		0.581	1.044	0.895–1.218		0.751	0.933	0.606–1.435	
SATF (g)	0.811	0.984	0.863–1.122		0.872	1.011	0.883–1.158		0.076	1.612	0.951–2.732	
MUFA (g)	0.854	1.011	0.901–1.134		0.862	0.989	0.873–1.120		0.144	1.361	0.900-2.058	
PUFA (g)	0.574	1.035	0.919–1.165		0.742	1.021	0.902–1.155		0.232	1.316	0.839–2.065	
SOD (g)	0.003	1.000	1.000-1.001		0.003	1.000	1.000-1.001		0.378	1.000	0.999-1.000	
Iron (mg)	0.361	0.964	0.890–1.044		0.950	1.005	0.869–1.162		0.364	0.841	0.578–1.223	
Mg (mg)	0.009	0.992	0.987–0.998		0.405	0.998	0.992–1.003		0.645	1.003	0.990–1.016	
Vit D (Ug)	0.006	1.406	1.105–1.788		0.044	1.298	1.007–1.674		0.955	0.981	0.501–1.922	
Vit C (mg)	0.036	1.006	1.000-1.012		0.218	1.004	0.998–1.010		0.722	0.997	0.982–1.013	
Vit A (RE)	0.149	0.999	0.997-1.000		0.138	0.999	0.997-1.000		0.206	0.997	0.993–1.002	

**Table 5:** Association between eating disorders and PMS

PMS symptoms		With an eating disorder (*N*)	No eating disorder (*N*)	*p* Value
Psychological symptoms	never	0% (0)	100% (7)	0.1
	mild	10% (7)	90% (63)	
	moderate	6.4% (7)	93.6% (103)	
	severe	18.6% (8)	81.4% (35)	
Physical symptoms	never	0 (0%)	100% (4)	0.1
	mild	4.5% (4)	95.5% (85)	
	moderate	12% (4)	88% (103)	
	severe	19% (4)	81% (17)	
Behavioral symptoms	never	4.5% (1)	95.5% (21)	0.8
	mild	10.2% (9)	89.8% (79)	
	moderate	9.8% (11)	90.2% (101)	
	severe	12.5% (1)	87.5% (7)	

## Discussion

Dietary intake appears to play a crucial role in managing PMS symptoms. However, the actual impact of dietary factors on PMS has not been extensively studied, and comprehensive research is needed to establish sufficient evidence supporting their use as an effective treatment strategy. The findings of this study contribute to a better understanding of the complex relationships between dietary factors and PMS symptoms, providing valuable insights for both clinical and lifestyle interventions aimed at symptom management.

This study identified a significant positive correlation between sodium and vitamin D intake and both psychological and physical symptoms of PMS. Additionally, vitamin C intake was positively associated with psychological symptoms. In contrast, magnesium intake demonstrated a significant negative association with psychological symptoms, suggesting a potential protective effect. Notably, behavioral symptoms exhibited marginal associations with dietary components such as caloric intake, saturated fat, and carbohydrate intake; however, these associations did not reach statistical significance. These findings underscore the need for further research to elucidate the role of dietary factors in PMS symptom modulation and to explore potential dietary interventions for symptom relief.

Since inflammation and oxidative stress contribute to the development of PMS, the consumption of antioxidants and anti-inflammatory nutrients, such as vitamins D and C, as well as magnesium (Mg), may help alleviate symptoms. However, existing findings remain inconsistent [20]. A study conducted in Fujiana reported that individuals with a diet high in Mg experienced an improvement in PMS symptoms [21]. Additionally, research has suggested that Mg supplementation may be effective in preventing dysmenorrhea, PMS, and menstrual migraines [22]. One study found that women who regularly consumed 250 mg of Mg supplements per day experienced a reduction in PMS symptom severity [23]. Furthermore, a recent clinical trial demonstrated that a combination of Mg and vitamin B6 could alleviate premenstrual anxiety [24]. However, findings on the association between Mg intake and PMS symptoms remain inconclusive. For instance, Erbil reported a weak positive correlation between PMS symptom severity scores and daily Mg intake among 219 nursing students in Turkey [25]. Magnesium is a vital mineral for the central nervous system, playing a crucial role in alleviating anxiety, irritability, and other psychological symptoms by regulating neurotransmitters such as gamma-aminobutyric acid (GABA), serotonin, and dopamine. These mechanisms enhance neuronal function and protect against excitotoxicity, which affects mood and cognition [26]. Additionally, Mg has been shown to reduce systemic inflammation by lowering cytokine levels and oxidative stress. Its role in regulating blood sugar levels and energy metabolism further underscores its potential to improve PMS-related symptoms [27]. Given that PMS symptoms such as mood swings, fatigue, and fluid retention closely resemble symptoms of Mg deficiency, it has been hypothesized that inadequate Mg levels may contribute to PMS pathophysiology [28].

In a 2023 study conducted by Farpour involving 125 Iranian women aged 20 to 46 years, it was found that unhealthy dietary patterns, specifically the Western diet characterized by high levels of fat and sodium, can significantly exacerbate PMS symptoms. This effect is attributed to disruptions in the balance of the antioxidant system, increases in inflammation, and elevated blood estrogen levels [29]. Similarly, a study conducted by Hashem (2019) among students aged 18 to 24 at the University of Sharjah identified the consumption of high-calorie foods, and sodium as risk factors for the physical symptoms associated with PMS [30]. Furthermore, research by Thakur demonstrated a correlation between high salt intake and PMS symptoms in a sample of 330 Indian women aged 18 to 24 [31]. These findings align with our results, which indicate that sodium intake is associated with the worsening of both physical and psychological symptoms of PMS. It appears that a high intake of dietary sodium may contribute to increased fluid retention. This phenomenon occurs because estrogen stimulates the liver to produce angiotensinogen, which subsequently enhances the release of aldosterone. Consequently, this hormonal response may lead to symptoms such as bloating and breast tenderness commonly associated with PMS [27].

Vitamin D plays a crucial role in influencing sex hormone levels, neurotransmitter function, and the reduction of inflammation, underscoring its significance in female reproductive health [32]. In this context, a study conducted by Abdollahi et al. demonstrated that vitamin D supplementation (200,000 IU administered every other day for 12 weeks) alleviated symptoms such as nervousness, fatigue, and reduced social and occupational functioning in young girls with PMS. However, this supplementation did not result in significant improvements in other PMS symptoms [33]. Conversely, research by Robinson et al. concluded that the existing evidence does not adequately support the efficacy of vitamin D in alleviating PMS in women [34]. Nonetheless, our findings suggest that vitamin D intake has a beneficial impact on both psychological and physical symptoms associated with PMS.

It is important to note that excessive vitamin D intake can lead to hypercalcemia, which may exacerbate symptoms including difficulties in concentration, confusion, depression, vomiting, and abdominal pain [35]. Additionally, vitamin D is involved in the regulation of the hypothalamus-pituitary-adrenal (HPA) axis, where any dysregulation can heighten stress sensitivity and contribute to mood disturbances [35]. Furthermore, variability in vitamin D metabolism, influenced by genetic polymorphisms or pre-existing health conditions, adds a layer of complexity to its effects [36].

The significant positive association observed between vitamin C intake and psychological symptoms of PMS in this study suggests a complex relationship that merits further investigation. Vitamin C is widely recognized for its antioxidant properties, which can help mitigate oxidative stress and support hormonal balance [37]. However, its role in PMS appears to be multifaceted, and several mechanisms may partially elucidate the observed exacerbation of symptoms.

Increased vitamin C intake may affect adrenal gland function, leading to heightened cortisol production, which can increase sensitivity to stress and contribute to mood fluctuations such as anxiety and irritability [38]. Moreover, vitamin C’s involvement in norepinephrine synthesis is crucial for energy and mood regulation; however, this may also exacerbate psychological symptoms in individuals predisposed to stress-related conditions [39].

In contrast to our findings, a study conducted by Ghazawi (2023) reported no correlation between menstrual cycle symptoms and the intake of magnesium, sodium, and vitamin C among adult women [40]. Nevertheless, research by Zeitoun et al. (2023) indicated that higher vitamin C intake is associated with increased premenstrual appetite changes and bloating in females aged 20 to 29 (*n* = 555) from the Toronto Nutrigenomics and Health Study [41]. The inconsistent findings regarding vitamin D and C in relation to PMS may stem not only from methodological differences, such as dietary assessment tools and baseline nutrient status, but also from individual variations in absorption, metabolism, and genetic polymorphisms. For instance, the effectiveness of vitamin D may be influenced by VDR gene variants, which affect its activity in the central nervous system [36]. Similarly, vitamin Cs dual role as both an antioxidant and a co-factor in stress-related pathways (e.g., cortisol and norepinephrine production) could explain why its effects differ across populations [38, 39]. These complex biological interactions highlight the need for further mechanistic and longitudinal studies to better clarify the role of these vitamins in PMS.

Erbil et al.‘s 2024 study found that the intake of certain nutrients influences the severity of PMS symptoms. They reported a weak positive correlation between PMS scores and daily intake of energy, and magnesium (Mg) among 219 nursing students in Turkey. However, no correlation was found between protein consumption and PMS, which aligns with our findings [25]. Additionally, Farpour et al. also reported no association between energy, macronutrient, and micronutrient intake and PMS among Iranian women aged 20 to 46 years [29].

In our study, only behavioral symptoms exhibited a marginal association with calorie, saturated fat, and carbohydrate intake, although these associations did not reach statistical significance. Research suggests that fats and saturated fatty acids may contribute to inflammation by elevating levels of inflammatory cytokines and C-reactive protein (CRP), potentially exacerbating PMS symptoms, including depression [42]. Concerning this, Ghazawi et al. (2023) found that saturated fat consumption increased the risk of psychological symptoms related to the menstrual cycle among Jordanian women [40].

A clinical trial by Ismail Pour et al. found that higher whole grain intake reduced PMS severity. This effect may be related to nutrients like magnesium, which support neurotransmitter regulation, including serotonin and gamma-aminobutyric acid (GABA) [43]. While some earlier studies have suggested possible links, current findings remain inconclusive, and further investigation is needed.

This study also revealed that individuals with eating disorders exhibited more severe symptoms of PMS compared to those without eating disorders, although these differences did not reach statistical significance. An epidemiological survey involving 8,694 participants in the United States found no significant association between PMS or premenstrual dysphoric disorder (PMDD) and binge-eating disorder, highlighting the complexity of their potential interplay [44]. In contrast, Yoshinari et al. (2024) found that eating behaviors significantly affected PMS symptoms in Japanese female college students, with those having eating disorders reporting more severe physical and psychological symptoms than those without [12].

Hormonal fluctuations throughout the menstrual cycle can adversely affect appetite regulation and eating behaviors in women. Increased food cravings prior to menstruation may lead to overeating, which can exacerbate existing eating disorders [44]. Nevertheless, our results do not confirm a definitive link, and further research with larger sample sizes is needed to explore this complex relationship.

This study has several limitations. Firstly, we employed the 3-day dietary recall method to assess dietary intake, which inherently relies on participants’ memory and may lead to both underreporting and overreporting of food consumption, potentially affecting the accuracy of the data obtained. Additionally, the full range of dietary components was not evaluated due to constraints such as the complexity of analyzing multiple nutrients and the feasibility of interpreting the data within the scope of this study.

Moreover, conducting this research online might result in less precise responses from participants, which could further limit the generalizability of the findings. Despite the limited sample size, the insights gained from this study contribute valuable information regarding the relationship between dietary factors and PMS. These results can serve as a foundation for more comprehensive and in-depth research in the future.

It is also important to note that this study utilized standardized tools, such as the Premenstrual Syndrome Screening Tool (PSST), along with strict inclusion and exclusion criteria, ensuring the selection of a relevant and focused sample. Given the heterogeneity observed in existing research, more rigorous studies are essential to ascertain whether dietary modifications can effectively alleviate PMS-related symptoms for many women worldwide.

## Conclusions

This study emphasizes the influence of specific dietary factors, including sodium, magnesium (Mg), vitamin C, and vitamin D, on PMS symptoms, particularly those related to psychological and physical health. Additionally, behavioral symptoms exhibited potential associations with calorie, saturated fat, and carbohydrate intake. Notably, individuals with eating disorders reported more severe PMS symptoms, underscoring the importance of dietary considerations in the management of PMS. In light of the findings, practical dietary recommendations include reducing sodium-rich foods, moderating vitamin D supplementation based on individual levels, and increasing intake of magnesium-rich foods such as leafy greens, legumes, and whole grains. Healthcare professionals may also consider screening for disordered eating when assessing PMS to offer more personalized dietary support. Further research in this area could provide valuable insights and contribute to the development of more effective treatment strategies for individuals experiencing PMS symptoms.

## Data Availability

The datasets used and/or analyzed during the current study available from the corresponding author on reasonable request. The entire dataset is in Farsi language. The Data can be available in English language for the readers and make available from the corresponding author on reasonable request.
